# Supplementary material for: Cell type-specific biotin labeling in vivo resolves regional neuronal and astrocyte proteomic differences in mouse brain
Source: Nat Commun. 2022 May 25;13:2927. doi: 10.1038/s41467-022-30623-x (PMC9132937; doi:10.1038/s41467-022-30623-x)
Supplement: Supplementary file 1 — Supplementary Information [file 41467_2022_30623_MOESM1_ESM.pdf]

SUPPLEMENTARY INFORMATION

Cell type-specific biotin labeling *in vivo* resolves regional neuronal and astrocyte proteomic differences in mouse brain

Sruti Rayaprolu[1,2], Sara Bitarafan[3], Juliet V. Santiago[1,2], Ranjita Betarbet[1,2], Sydney Sunna[1,2], Lihong Cheng[1,2], Hailian Xiao[1,2], Ruth S. Nelson[1,2], Prateek Kumar[1,2], Pritha Bagchi[2,4,5], Duc M. Duong[2,4,5], Annie M. Goettemoeller[6], Viktor János Oláh[6], Matt Rowan[6], Allan I. Levey[1,2], Levi B. Wood[3], Nicholas T. Seyfried[2,4,5]*, Srikant Rangaraju[1,2]*

Affiliations

[1]Department of Neurology, Emory University, Atlanta, GA 30322, USA

[2]Center for Neurodegenerative Diseases, Emory University, Atlanta, GA, 30322, USA

[3]Georgia W. Woodruff School of Mechanical Engineering, Parker H. Petit Institute for Bioengineering and Bioscience, and Wallace H. Coulter Department of Biomedical Engineering, Georgia Institute of Technology, Atlanta, GA, 30332 USA

[4]Emory Integrated Proteomics Core, Emory University, Atlanta, GA 30322, USA

[5]Department of Biochemistry, Emory University, Atlanta, GA 30322, USA

[6]Department of Cell Biology, Emory University, Atlanta, GA 30322, USA

SUPPLEMENTARY FIGURES

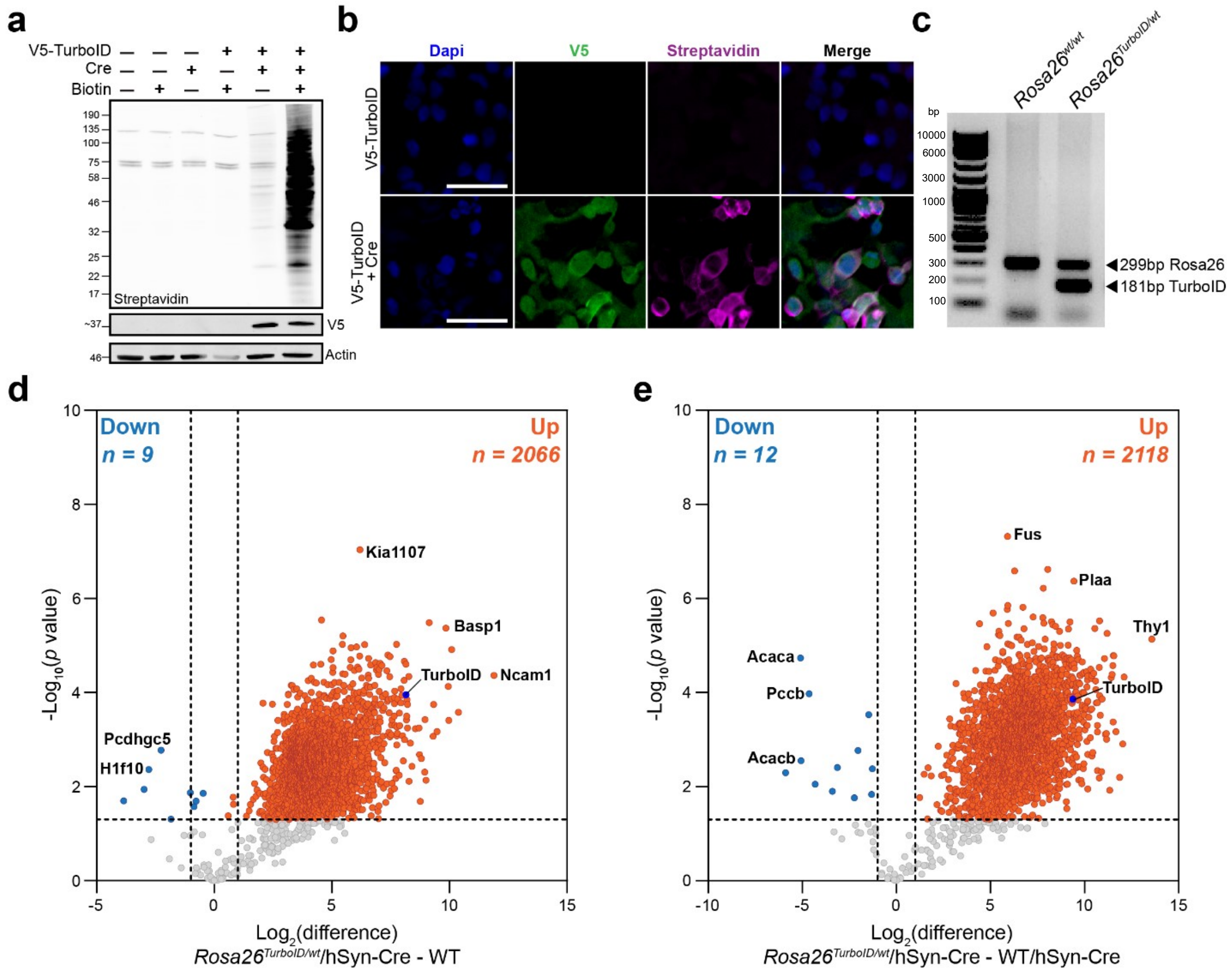

Supplementary Figure 1. *In vitro* validation of Rosa26 TurboID targeting vector. These data are associated with Figure 1**. a** Western blot studies of whole cell HEK293 lysates after transfection with Rosa26 TurboID targeting vector or sham, and co-transfection with CMV-Cre plasmid followed by biotin supplementation in culture media (*n* = 3 independent experiments per condition). Biotinylation (streptavidin680), V5 (to detect TurboID), and actin were measured. As compared to few endogenous biotinylated proteins in un-transfected or single plasmid *transfected* cells, only co-transfected cells treated with biotin showed biotinylation while low level biotinylation was also observed in biotin non-supplemented wells due to the presence of biotin in cell culture media. **b** Representative immunofluorescence images of cells (*n* = 3 independent experiments per condition) adhered to coverslips, from experiments in (a), confirming expression of V5-TurboID-NES (green) and biotinylation (magenta) in co-transfected HEK293 cells. Scale bar = 200 µm **c** Representative genotyping PCR results (*n* = 3 independent mice per group) obtained from *Rosa26*$^{wt/wt}$ and *Rosa26*$^{TurboID/wt}$ littermate mice. The 181bp band corresponds TurboID transgene and 299bp band represents endogenous *ROSA26* allele. **d, e** Volcano plots showing differentially expressed proteins comparing (d) *Rosa26*$^{TurboID/wt}$/hSyn-Cre with un-injected WT mice, and (e) *Rosa26*$^{TurboID/wt}$ /hSyn-Cre mice with WT/hSyn-Cre mice. Orange symbols (two-tailed T test unadjusted $p \leq 0.05$ and ≥ 2-fold change) represent biotinylated proteins enriched in the *Rosa26*$^{TurboID/wt}$/hSyn-Cre brains while blue symbols

represent biotinylated proteins enriched in control groups. For related MS data and additional analyses, see Supplementary Data 1, 7, & 8.

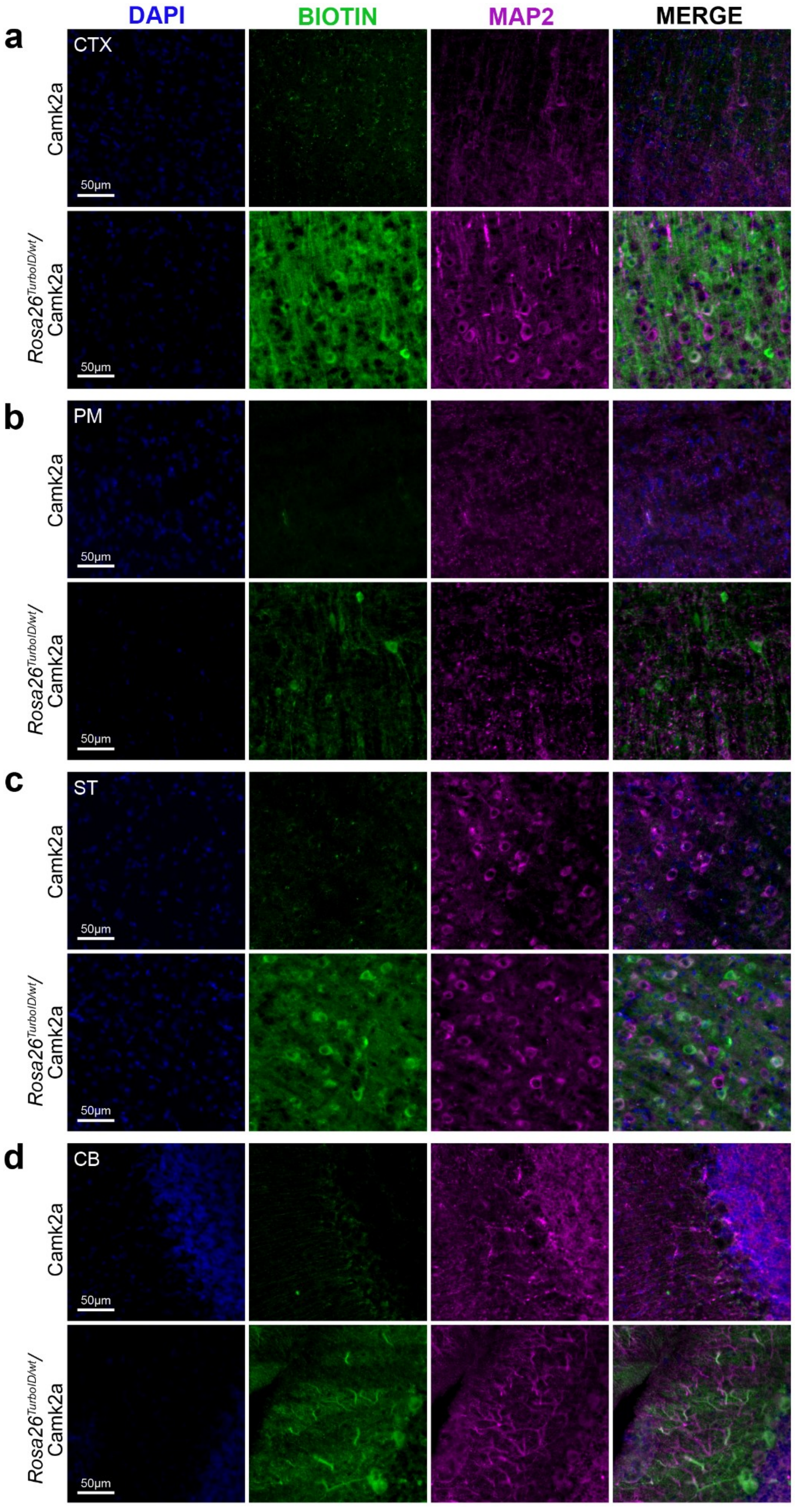

DAPI
BIOTIN
MAP2
MERGE
a
CTX
Camk2a
Rosa26TurboID/wt/ Camk2a
50µm
b
PM
c
ST
d
CB

Supplementary Figure 2. Immuno-histochemical confirmation of neuronal biotinylation in *Rosa26*$^{TurboID/wt}$/Camk2a mice. These data are associated with Figures 2 and 3. Representative immunofluorescence images (*n* = 3 mice per group) displaying robust biotinylation (green: streptavidin Alexa488) within neuronal cell bodies and axons (magenta: Map2) in the **a** cortex (CTX), **b** pons/medulla (PM), **c** striatum/thalamus (ST), and **d** cerebellum (CB). Degree of biotinylation varied by region, with highest biotinylation observed in the CTX and PM regions. Neurons labeled in the CTX were mostly pyramidal neurons while Purkinje neurons were labeled in the cerebellum. PM showed labeling predominantly of axons. Nuclei were labeled with DAPI (blue).

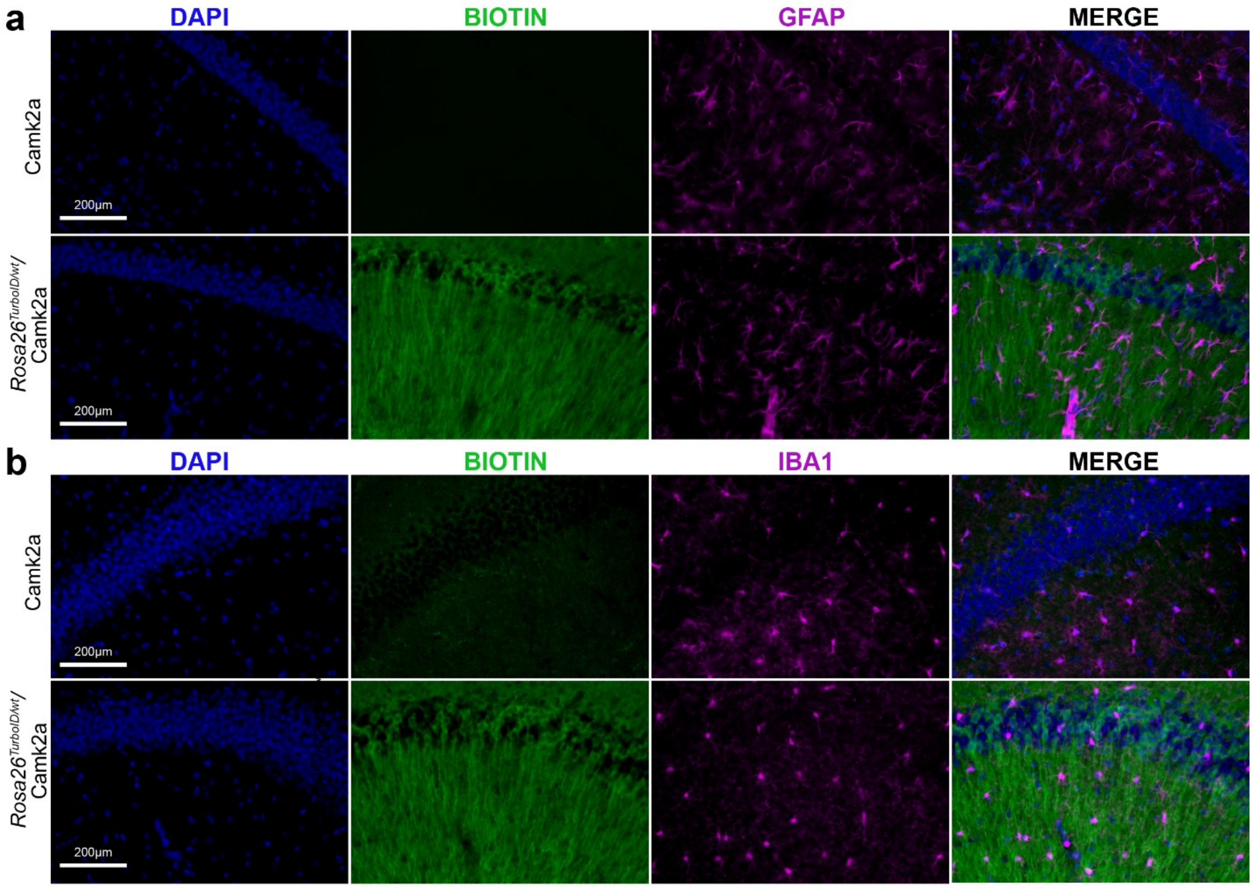

Supplementary Figure 3. Lack of glial cell biotinyation or reactive glial morphological changes in *Rosa26*$^{TurboID/wt}$/Camk2a mice. These data are associated with Figures 2 and 3. **a, b** Representative immunofluorescence images of CA2 of the hippocampus confirming lack of overlap between biotinylation (green: streptavidin Alexa488) with **a** astrocytes (magenta: Gfap) or **b** microglia (magenta: Iba1). Nuclei were labeled with DAPI (blue) (*n* = 2 mice per experimental group.)

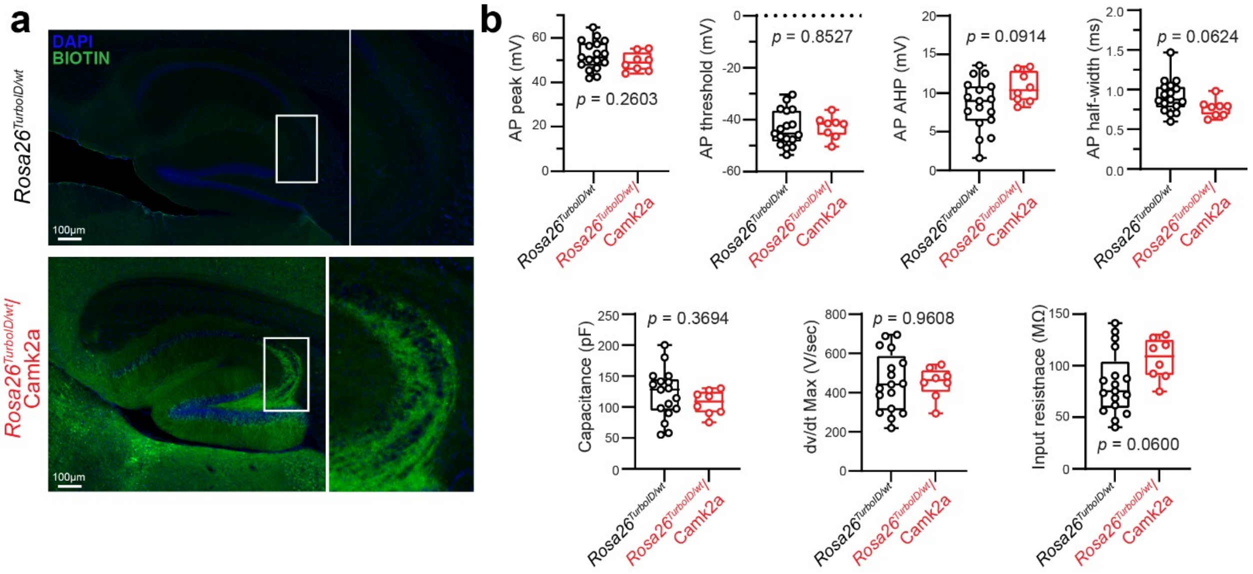

Supplementary Figure 4. Lack of electrophysiological alterations in hippocampal CA3 pyramidal neurons in *Rosa26*$^{TurboID/wt}$/Camk2a mice. These data are associated with Figure 2. **a** Representative immunofluorescence images of the hippocampus from control (*Rosa26*$^{TurboID/wt}$) and *Rosa26*$^{TurboID/wt}$/Camk2a-Cre^{Ert2} displaying biotinylation within cells, specifically CA3 neurons. **b** Summary data from whole-cell current clamp recordings in CA3c in control (*Rosa*$^{TurboID/wt}$) and labeled *Rosa26*$^{TurboID/wt}$/Camk2a-Cre^{Ert2} mice. Each data point represents a single neuron. Pooled analysis from n = 17 non-labeled control and n = 8 labeled neurons (n = 2 mice/group; p value in each graph represents unpaired t-test). Data are represented as box plots, indicating median, inter-quartile range, 10th and 90th percentile. Source data are provided as a Source Data file.

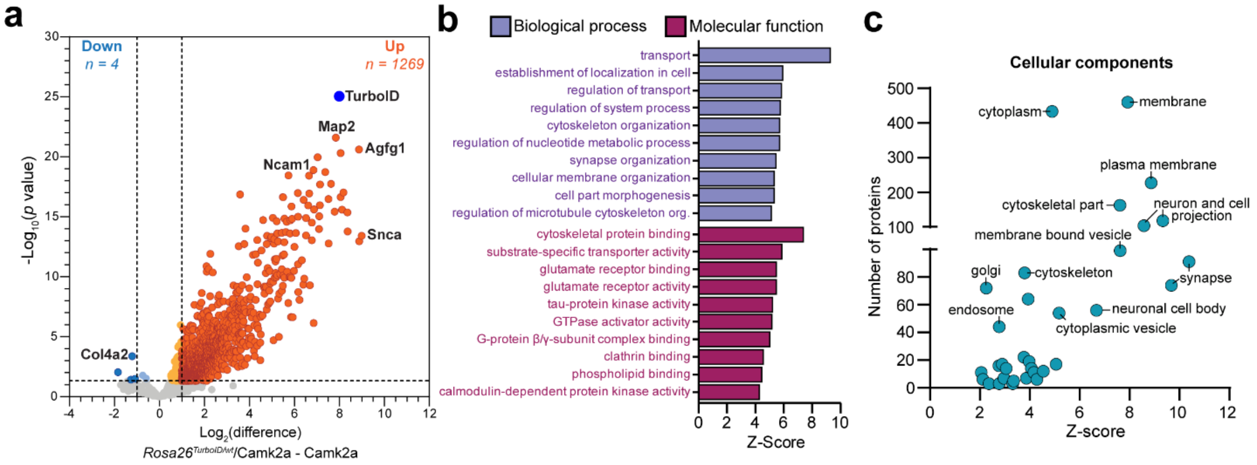

Supplementary Figure 5. *In vivo* Camk2a-positive neuronal proteomic biotinylation by TurboID provides a representative cellular proteome. a Volcano plot of MS data showing differentially enriched proteins between labeled *Rosa26*$^{TurboID/wt}$/Camk2a-Cre^{Ert2} and non-labeled Camk2a-Cre^{Ert2} control mouse brain. For this analysis, all 5 brain regions were combined for both groups. Orange symbols (two-tailed T test unadjusted $p \leq 0.05$ and ≥ 2-fold change) represent biotinylated proteins enriched in the *Rosa26*$^{TurboID/wt}$/Camk2a-Cre^{Ert2} brain while blue symbols represent biotinylated proteins enriched in control brain. **b** GSEA of biotinylated proteins (orange symbols in panel a) showed neuron-specific gene ontology terms as well as metabolic, cytoskeletal, ion transporter, and endocytosis related gene ontology terms. **c** Cellular component gene ontology terms enriched in Camk2a proteome showing several cellular sub-compartments and organelles as well as the synapse. For related MS data and additional analyses, see Supplementary Data 9 & 10.

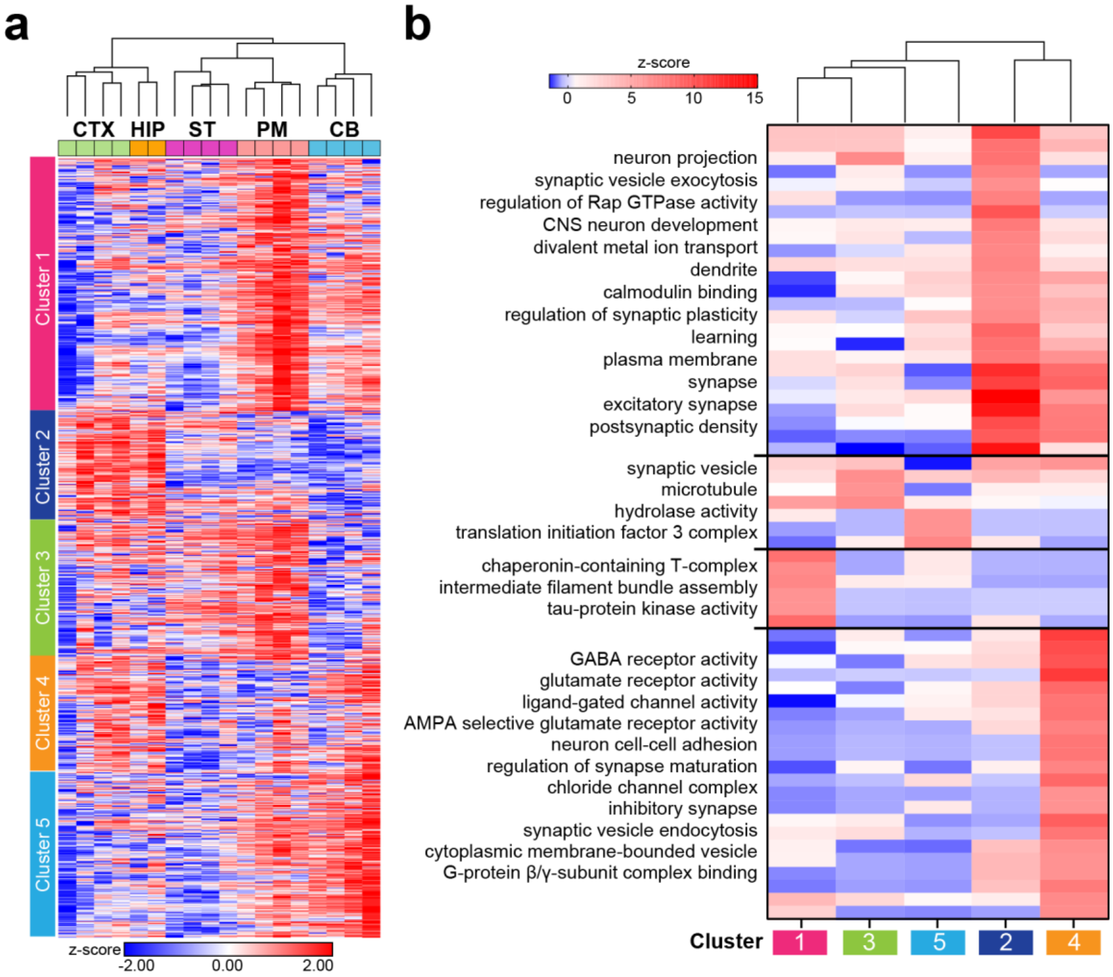

Supplementary Figure 6. Region-specific proteomic differences in adult mouse Camk2a. **a** Unsupervised hierarchical clustering analysis (HCA) of 1,245 Camk2a neuronal proteins (based on rows and columns) from *Rosa26$^{TurboID/wt}$*/Camk2a-Cre^{Ert2} mice showed distinct clusters of proteins, primarily highlighting region-specific patterns of protein expression. Prior to HCA, MS data was normalized to TurboID abundance per sample to account for inherent differences in TurboID expression and/or Camk2a promoter activity across regions. Individual clusters are shown with distinct colors. **b** Heat map representation (HCA) of results from GSEA to identify over-represented molecular functions, cellular components, and biological processes within clusters shown in panel (a). Representative gene ontology terms are highlighted. For related MS data and additional analyses, see Supplementary Data 14 & 15.

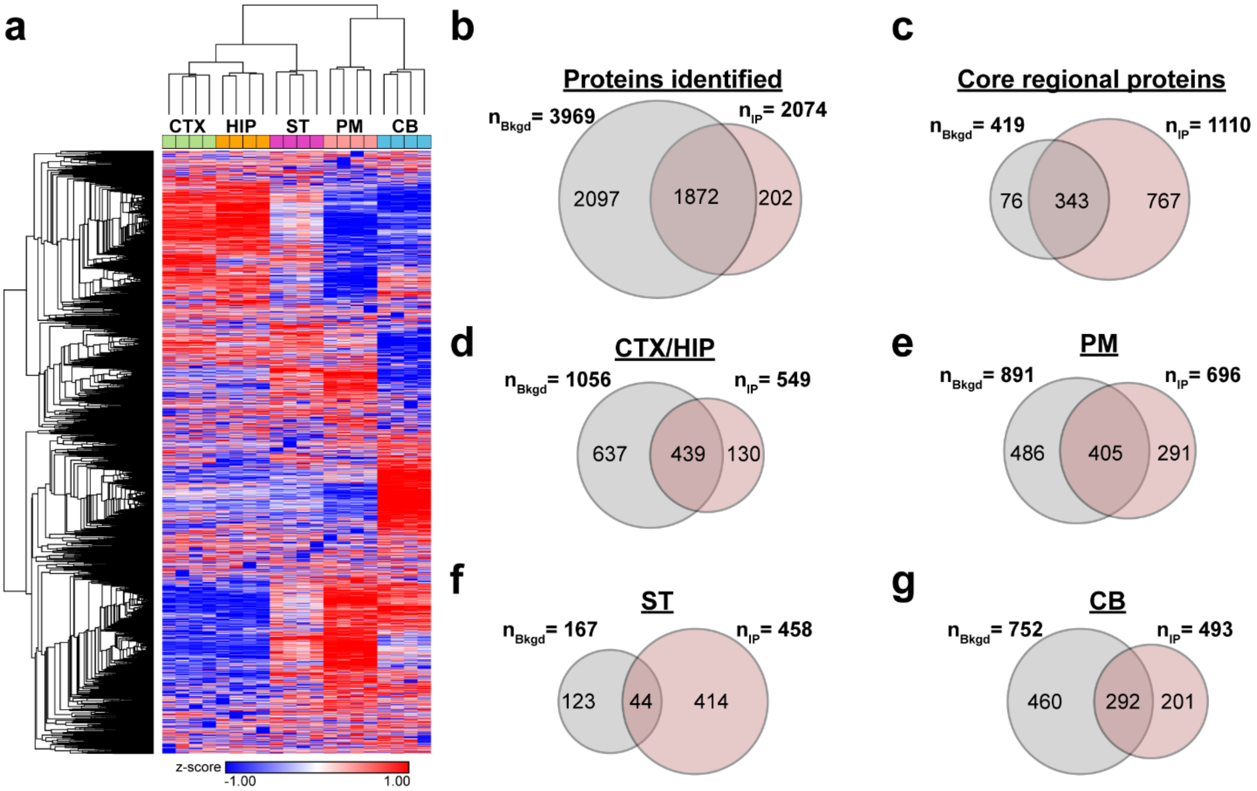

Supplementary Figure 7. **Camk2a-CIBOP proteome identifies twice as many core regional proteomic differences than total brain proteome**. **a** Unsupervised hierarchical clustering analysis (HCA) of 3,969 proteins (based on rows and columns) from *Rosa26*$^{TurboID/wt}$/Camk2a-Cre^{Ert2} and Camk2a-Cre^{Ert2} total brain lysates show distinct clusters of proteins, primarily highlighting region-specific patterns of protein expression. Total brain regional proteomes are detailed in Supplementary data 16. **b-g** Comparison of total brain the overlap between background (Bkgd) and *Rosa26*$^{TurboID/wt}$/Camk2a (IP) labeled proteomes (b), core regional proteins (**c**) defined by ≥4-fold enrichment in the specific region over other regions and $p \leq 0.05$), and each region: cortex/hippocampus (CTX, **d**), pons/medulla (PM, **e**), striatum/thalamus (ST, **f**), cerebellum (CB, **g**).

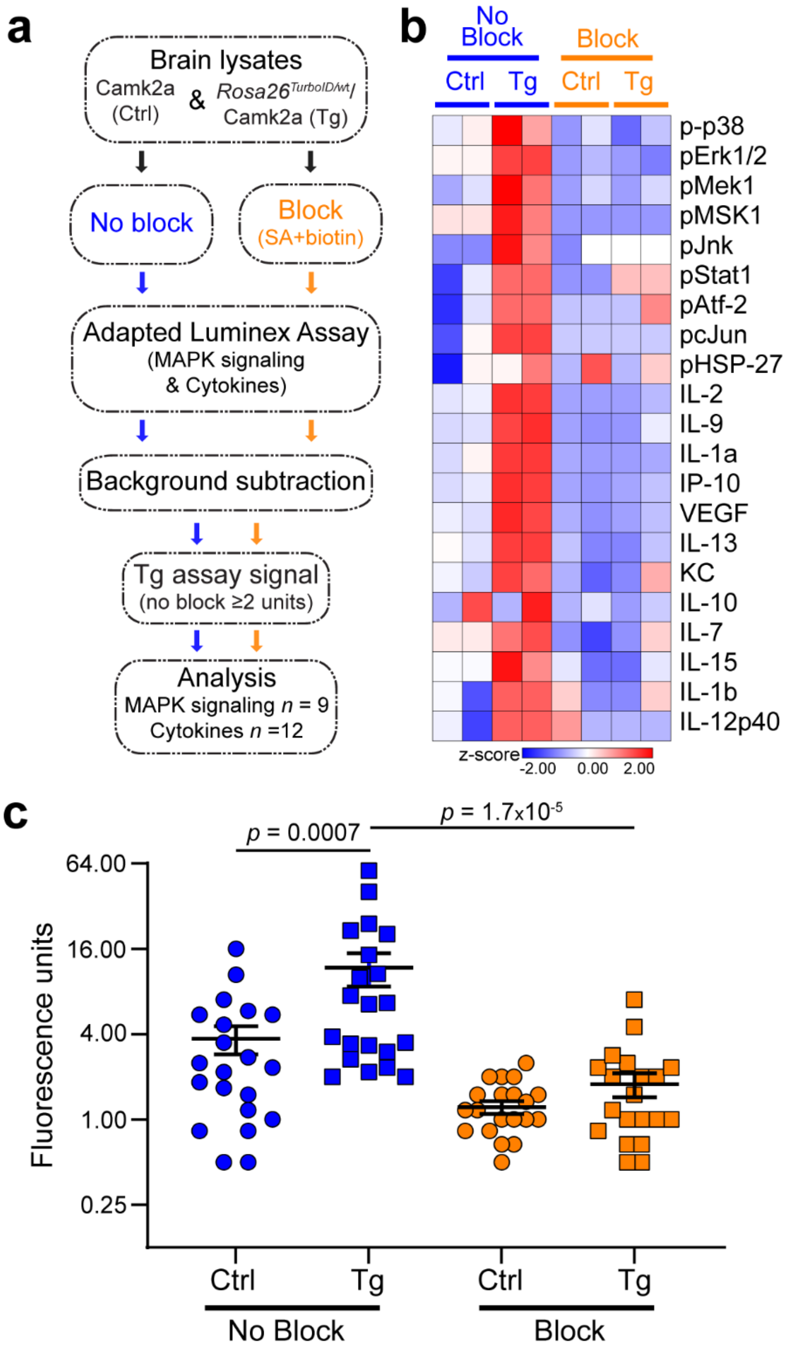

Supplementary Figure 8. Validation of adapted Luminex assay. a Workflow detailing blocking experiments with streptavidin and biotin (SA+biotin) using brain lysates from Camk2a control (ctrl) and *Rosa*$^{TurboID/wt}$/Camk2a labeled (Tg) mice. **b** Heat map showing results from normalized data from adapted Luminex assay with or without blocking with SA+biotin. Phospho-proteins from MAPK pathway and cytokines are shown. **c** Fluorescence values (after background subtraction) for MAPK phospho-proteins and cytokines in brain ctrl and Tg brain lysates (n=21 analytes measured per sample, two-tailed T test unadjusted p values are shown). Data are presented as means ± standard error of mean (SEM). There is a higher signal in the

Tg mice compared to the control and a loss of this signal when blocked with SA+biotin, confirming that we are indeed detecting TurboID biotinylated neuronal phospho-proteins and cytokines in the brain. Source data are provided as a Source Data file.

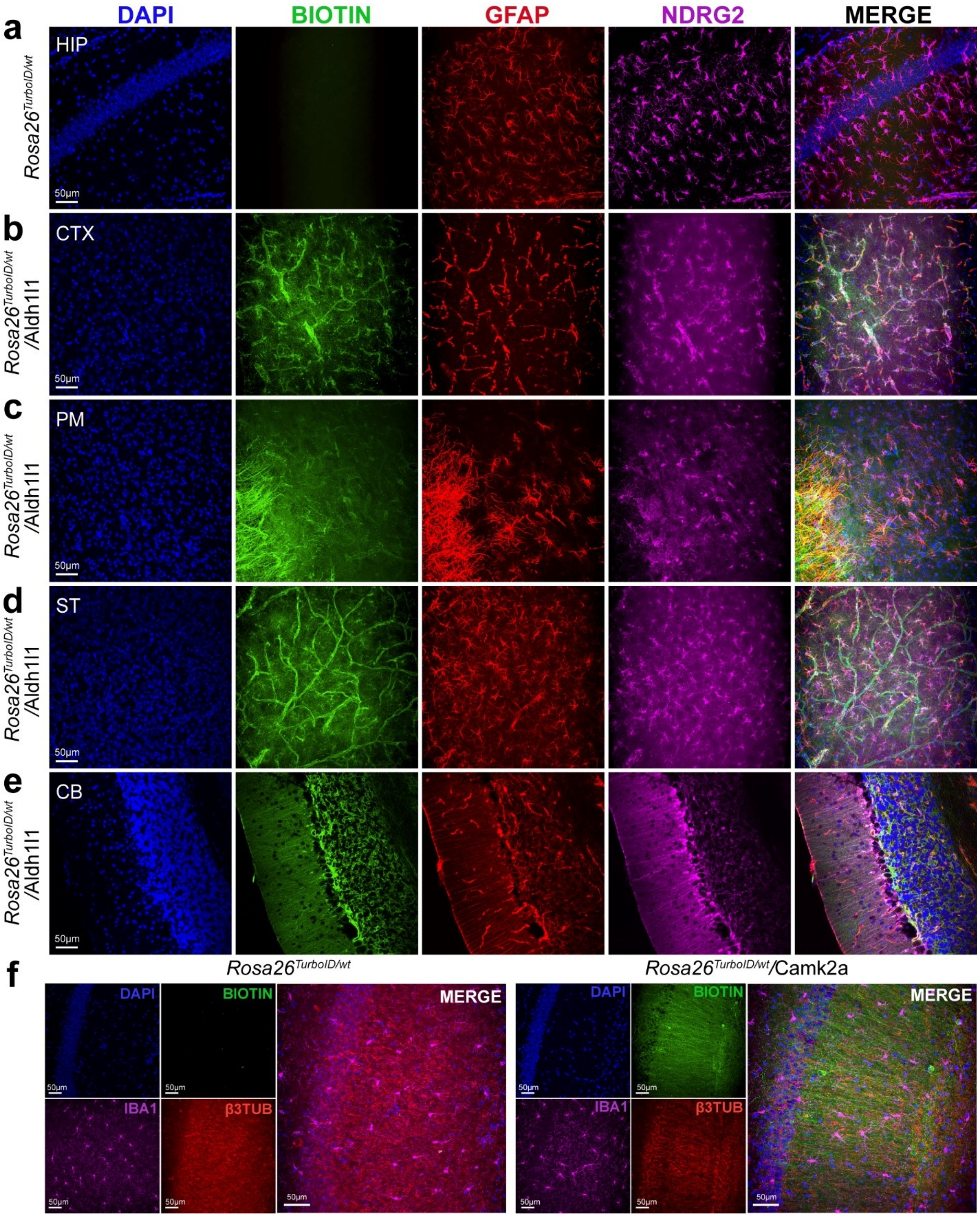

Supplementary Figure 9. Immuno-histochemical confirmation of astrocytic biotinylation in *Rosa26*$^{TurboID/wt}$/Aldh1l1 mice. These data are associated with Figure 5. Representative immunofluorescence images displaying robust biotinylation (green: streptavidin Alexa488) within astrocytes (red: Gfap; magenta: Ndrg2) and neighboring blood vessels in **a** control hippocampus, **b** Rosa26TurboID/wt/Aldh1l1 cortex (CTX), **c** pons/medulla (PM), **d**

striatum/thalamus (ST), and **e** cerebellum (CB). **f** Confirmation of neuronal biotinylation (green: streptavidin Alexa488; red: βIII-Tubulin) in *Rosa26*$^{TurboID/wt}$/Camk2a hippocampus and absence of microglial labeling or activation (magenta: Iba1) compared to control hippocampus. Nuclei were labeled with DAPI (blue) (n = 2 mice per experimental group).

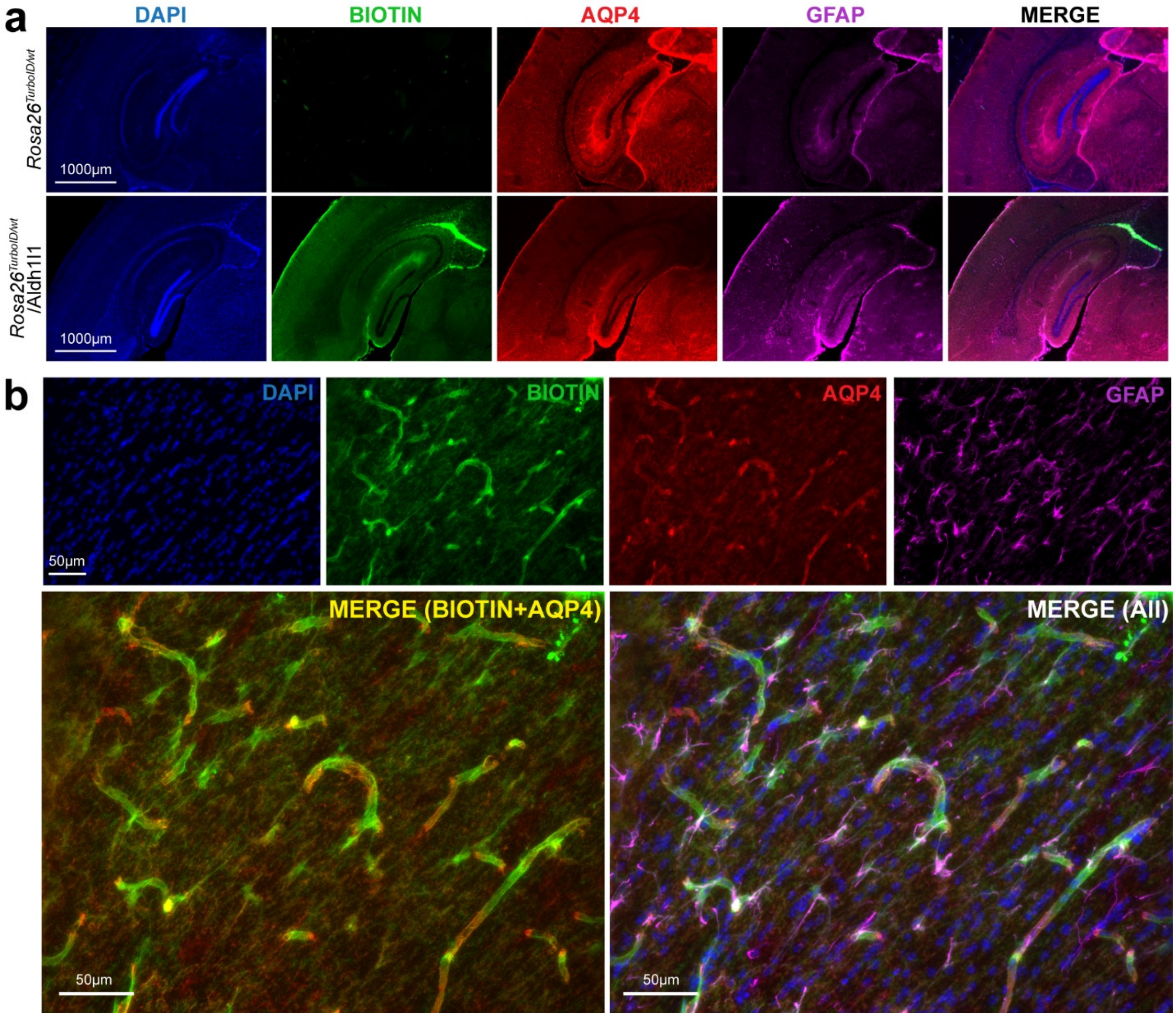

Supplementary Figure 10. Aquaporin 4-positive astrocytic end-feet biotinylation in *Rosa26$^{TurboID/wt}$*/Aldh1l1 mice. a Representative immunofluorescence images (*n* = 2 mice per experimental group) displaying robust biotinylation (green: streptavidin Alexa488) within *Rosa26$^{TurboID/wt}$*/Aldh1l1 hippocampus, compared to *Rosa26$^{TurboID/wt}$* control hippocampus, and co-immunolabeling of AQP4 (red) positive astrocytic end-feet and Gfap (magenta) positive astrocytes. **b** Higher magnification images (*n* = 2 mice per experimental group) displaying the colocalization of biotin labeling with AQP4 positive astrocytic end-feet and Gfap positive astrocytes in *Rosa26$^{TurboID/wt}$*/Aldh1l1 hippocampus.

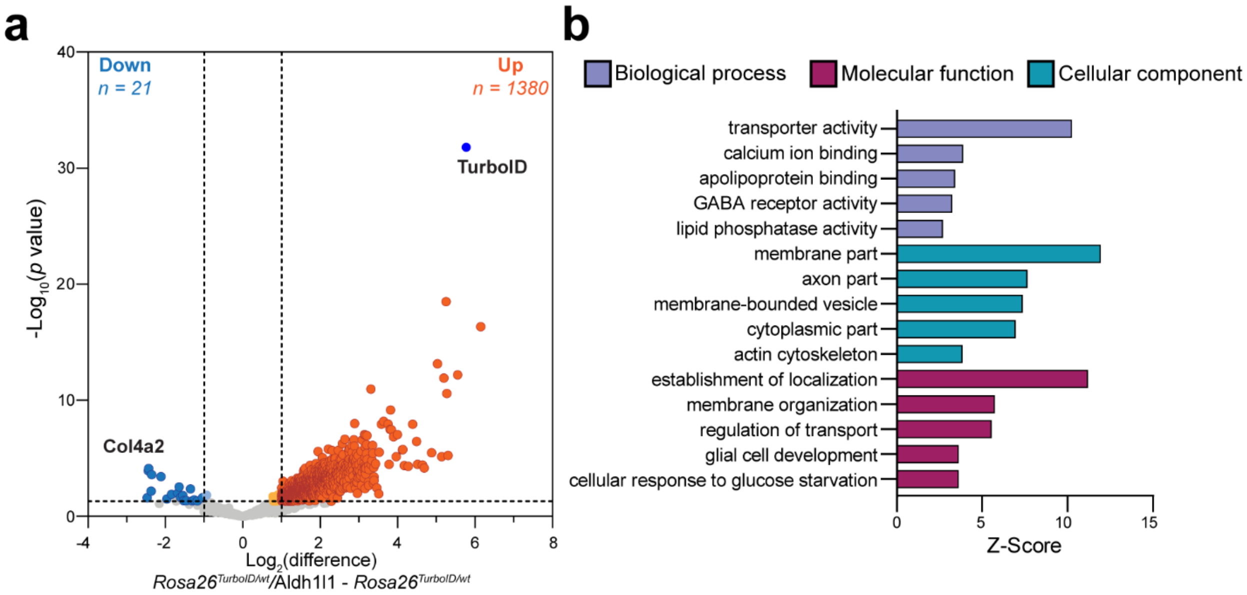

Supplementary Figure 11. *In vivo* protein biotinylation by TurboID captures Aldh1l1-positive astrocyte proteome. a Volcano plot of MS data showing differentially enriched proteins between labeled *Rosa26*$^{TurboID/wt}$/Aldh1l1-Cre^{Ert2} and non-labeled *Rosa26*$^{TurboID/wt}$ control mouse brain. For this analysis, all 6 brain regions were combined for both groups. Orange symbols (two-tailed T test unadjusted $p \leq 0.05$ and ≥ 2-fold change) represent biotinylated proteins enriched in the *Rosa26*$^{TurboID/wt}$/Aldh1l1-Cre^{Ert2} brain while blue symbols represent biotinylated proteins enriched in control brain. **b** GSEA of astrocyte enriched biotinylated proteins (orange symbols in panel a). For related MS data and additional analyses, see Supplementary Data 19 & 20.
