# Supplementary material for: Cell type-specific biotin labeling in vivo resolves regional neuronal and astrocyte proteomic differences in mouse brain
Source: Nat Commun. 2022 May 25;13:2927. doi: 10.1038/s41467-022-30623-x (PMC9132937; doi:10.1038/s41467-022-30623-x)
Supplement: Supplementary file 3 — Description of Additional Supplementary Files [file 41467_2022_30623_MOESM3_ESM.docx]

**Description of Additional Supplementary Files**

File name: Supplementary Data 1

Description: Log2 transformed protein abundances and differential enrichment analyses from RosaTurboID/wt AAV cohort. These data are associated Figure 1f volcano plot. P values are from T-test (2 tailed, assuming equal variance). Benjamin-Hochberg false-discovery-rate (BH-FDR) p values are also shown.

File name: Supplementary Data 2

Description: Log_2_ transformed phospho-peptide abundances and differential enrichment analyses of Rosa^TurboID/wt^ AAV cohort.

File name: Supplementary Data 3

Description: GSEA analysis of differentially enriched proteins (n = 2143) from RosaTurboID/wt+hSyn vs. (WT & WT+hSyn). These data are associated Figure 1f-h

File name: Supplementary Data 4

Description: Selected biotinylated cell surface, transporter, and ion channel proteins in neurons from the AAV cohort.

File name: Supplemental Table 5

Description: Log2 transformed protein abundances from total brain lysates from RosaTurboID/wt AAV cohort.

File name: Supplementary Data 6

Description: GSEA analysis of neuronal enriched biotinylated proteins (n = 354) not identified in total brain proteome from AAV cohort.

File name: Supplementary Data 7

Description: GSEA analysis of differentially enriched proteins (n = 2066) from RosaTurboID/wt+hSyn vs. WT. These data are associated with Supplemental Figure 1d

File name: Supplementary Data 8

Description: GSEA analysis of differentially enriched proteins (n = 2118) from RosaTurboID/wt+hSyn vs. WT+hSyn. These data are associated with Supplemental Figure 1e

File name: Supplementary Data 9

Description: Log2 transformed protein abundances and differential enrichment analyses from RosaTurboID/wt transgenic cohort 1. These data are associated with Supplemental Figure 5a. P values are from T-test (2 tailed, assuming equal variance). Benjamin-Hochberg false-discovery-rate (BH-FDR) p values are also shown.

File name: Supplementary Data 10

Description: GSEA analysis of differentially enriched proteins (n = 1245) from RosaTurboID/wt/Camk2a-CreErt2 vs. Camk2a-CreErt2. These data are associated with Supplemental Figure 5b-c

File name: Supplementary Data 11

Description: Core brain regional proteomic signatures of Camk2a positive neurons from RosaTurboID/wt transgenic cohort 1. This table is associated with Fig 3c. P values are from T-test (2 tailed, assuming equal variance).

File name: Supplementary Data 12

Description: GSEA of Camk2a core regional signature proteins. This table is associated with Figure 3c-d

File name: Supplementary Data 13

Description: Summary of protein/gene-disease associations of core brain regional proteomic signatures of Camk2a positive neurons

File name: Supplementary Data 14

Description: Summary of K-means clusters and abundance values of Camk2a regional proteome. This table is associated with Supplemental Figure 6a.

File name: Supplementary Data 15

Description: GSEA analysis of K-means clusters from RosaTurboID/wt/Camk2a-CreErt2 brain regions. These data are associated with Supplemental Figure 6b.

File name: Supplementary Data 16

Description: Log2 transformed protein abundances from whole brain (background) lysates from RosaTurboID/wt/Camk2a-CreErt2 Tg cohort 1. These data are associated with Supplemental Figure 7.

File name: Supplementary Data 17

Description: Luminex Phospho-signaling and cytokine assays of total brain lysates from AAV Cohort. These data are associated with Figure 4c-e. P values are from T-test (2 tailed, assuming equal variance)

File name: Supplementary Data 18

Description: Luminex analyses of brain lysates from Camk2a-CreErt2/Rosa26TurboID/wt mice: Regional analysis. These data are associated with Figure 4g-h. P values are from T-test (2 tailed, assuming equal variance)

File name: Supplementary Data 19

Description: Log2 transformed protein abundances and differential enrichment analyses from RosaTurboID/wt transgenic cohort 2. These data are associated with Supplemental Figure 8a and 5h. P values are from T-test (2 tailed, assuming equal variance). Benjamin-Hochberg false-discovery-rate (BH-FDR) p values are also shown.

File name: Supplementary Data 20

Description: GSEA analysis of differentially enriched proteins (n = 1380) from RosaTurboID/wt/Camk2a-CreErt2 vs. Camk2a-CreErt2. These data are associated with Supplemental Figure 8b

File name: Supplementary Data 21

Description: Core brain regional proteomic signatures of Aldh1l1 positive astrocytes from RosaTurboID/wt transgenic cohort 2. This table is associated with Fig 5f. P values are from T-test (2 tailed, assuming equal variance).

File name: Supplementary Data 22

Description: GSEA of Aldh1l1 core regional signature proteins. This table is associated with Figure 5g

File name: Supplementary Data 23

Description: GSEA analysis of differentially enriched proteins from RosaTurboID/wt/Camk2a-CreErt2 vs. RosaTurboID/wt/Aldh1l1-CreErt2. These data are associated with Figure 5i

File name: Supplementary Data 24

Description: Luminex analyses of brain lysates from Camk2a-CreErt2/Rosa26TurboID/wt and Aldh1l1-CreErt2/Rosa26TurboID/wt mice: Regional analysis. These data are associated with Figure 5j. P values are from T-test (2 tailed, assuming equal variance).
